# Active case detection methods for crusted scabies and leprosy: A systematic review

**DOI:** 10.1371/journal.pntd.0009577

**Published:** 2021-07-23

**Authors:** Miriam Glennie, Karen Gardner, Michelle Dowden, Bart J. Currie

**Affiliations:** 1 Public Sector Research Group, University New South Wales, Canberra, Australia; 2 One Disease, Darwin, Australia; 3 Menzies School of Health Research, Charles Darwin University and Royal Darwin Hospital, Darwin, Australia; Kwame Nkrumah University of Science and Technology, GHANA

## Abstract

**Background:**

Crusted scabies is endemic in some remote Aboriginal communities in the Northern Territory (NT) of Australia and carries a high mortality risk. Improvement in active case detection (ACD) for crusted scabies is hampered by a lack of evidence about best practice. We therefore conducted a systematic review of ACD methods for leprosy, a condition with similar ACD requirements, to consider how findings could be informative to crusted scabies detection.

**Methods and principle findings:**

We conducted systematic searches in MEDLINE, CINAHL, Scopus and the Cochrane Database for Systematic Reviews for studies published since 1999 that reported at least one comparison rate (detection or prevalence rate) against which the yield of the ACD method could be assessed. The search yielded 15 eligible studies from 511. Study heterogeneity precluded meta-analysis. Contact tracing and community screening of marginalised ethnic groups yielded the highest new case detection rates. Rapid community screening campaigns, and those using less experienced screening personnel, were associated with lower suspect confirmation rates. There is insufficient data to assess whether ACD campaigns improve treatment outcomes or disease control.

**Conclusion:**

This review demonstrates the importance of ACD campaigns in communities facing the highest barriers to healthcare access and within neighbourhoods of index cases. The potential benefit of ACD for crusted scabies is not quantified, however, lessons from leprosy suggest value in follow-up with previously identified cases and their close contacts to support for scabies control and to reduce the likelihood of reinfection in the crusted scabies case. Skilled screening personnel and appropriate community engagement strategies are needed to maximise screening uptake. More research is needed to assess ACD cost effectiveness, impact on disease control, and to explore ACD methods capable of capturing the homeless and highly mobile who may be missed in household centric models.

## Author summary

Crusted scabies is a severely debilitating skin disease with potentially fatal consequences that is endemic in some remote Aboriginal communities in the Northern Territory (NT) of Australia. Early detection is essential to maximise the chance of recovery and help prevent transmission of scabies to others. There is little knowledge about how to actively detect crusted scabies, so we sought relevant knowledge about a disease with many similarities: leprosy. The review demonstrates the importance of ACD campaigns in communities facing the highest barriers to healthcare access and within neighbourhoods of index cases. Lessons for crusted scabies in the NT include the importance of skilled screening personnel and appropriate community engagement strategies to maximise screening uptake. More research is needed to assess ACD cost effectiveness, impact on disease control, and to explore ACD methods capable of capturing the homeless and highly mobile who may be missed in household centric models.

## Introduction

Caused by *Sarcoptes scabiei*, the same mite that causes simple scabies, crusted scabies is a severe, progressive and debilitating form of scabies that occurs in individuals with reduced immune competence to control mite replication [1]. In immunosuppressed individuals, exposure to simple scabies can lead to crusting of the skin due to mite loads of up to a million or more [2,3]. Secondary bacterial skin infections associated with scratching, can lead to bacteremia with sepsis and death, chronic lymphadenopathy, post-streptococcal glomerulonephritis and rheumatic heart disease, [4]. Historically crusted scabies had a 5-year mortality rate of up to 50% [5]. People with crusted scabies are highly infectious core transmitters of scabies, known to cause scabies outbreaks and contribute to scabies endemicity [3,6], making early detection of crusted scabies an important component of scabies control. This is particularly so given the increasing use of Mass Drug Administration (MDA) programs for scabies control in scabies endemic communities globally [6], as even a single crusted scabies case risks reducing MDA program efficacy and subsequently, acceptability [7].

Crusted scabies is endemic in some remote Aboriginal communities in the Northern Territory (NT) of Australia, and is more common when scabies is also endemic [8]. Remote Aboriginal communities carry an estimated crusted scabies prevalence rate of up to 24/10,000 [9] compared with an estimated rate of < 0. 1/10000 in the general Australian population (personal communication, B Currie). Stigma, high barriers to healthcare access, and poor clinical awareness due to its rarity in the general Australian population, all contribute to late stage diagnosis and high mortality rates [10].

In 2016, crusted scabies was upgraded to a notifiable disease in the NT, which creates an imperative for a more systematic approach to disease control [11]. Despite this heightened imperative and the clear barriers to healthcare access in remote Aboriginal communities, there is limited systematic use of active case detection (ACD) for crusted scabies. In comparison to passive case detection (PCD), which relies on patient self-report to a healthcare centre, ACD seeks out cases (e.g. in communities, households) to interrupt transmission and improve treatment outcomes. ACD is used widely in infectious disease elimination and control programs, including for skin-related neglected tropical diseases (NTDs), and is particularly important in populations facing high barriers to healthcare access such as poverty, marginalisation and/or disease stigma [12]. Campaign design and implementation impact the effectiveness of ACD, with personnel, logistics, and community buy-in being shown to be particularly important [13,14].

There is limited literature on ACD for crusted scabies, which creates a challenge for designing appropriate campaigns; to inform design considerations, this review sought to identify lessons from evidence based practice in leprosy. Leprosy is a skin NTD sharing many commonalities with crusted scabies from an ACD perspective, even while noting that leprosy is low infectivity and crusted scabies is highly infectious and at the extreme end of the clinical spectrum of scabies. Caused by the bacillus, *Mycobacterium leprae*, leprosy causes skin lesions and nerve damage which can progress to debilitating physical deformity [15,16]. Unlike crusted scabies, leprosy has long been a notifiable disease in most jurisdictions globally and is widely researched. While leprosy now has low prevalence (<1/10,000) in most tropical regions, pockets of endemicity remain in some countries [17], largely in communities marginalised by poverty, ethnicity, gender and/or age, and facing barriers to healthcare access [18,19,20]. ACD continues to play an important role in leprosy control in endemic regions [21].

Similarly to crusted scabies, leprosy is suitable for community-based skin screening by trained health workers, but requires specialist input and laboratory confirmation for diagnosis. Misdiagnosis of leprosy can occur in cases of fungal infections, and diagnosis should be confirmed through slit skin smear [22] or nucleic acid testing [23]. The specificity of clinical diagnosis by health workers is likely to be higher in crusted scabies than in leprosy, reflecting the greater clinical diversity across the spectrum of leprosy. However, misdiagnosis of crusted scabies can occur in cases of crusted skin sores and diagnosis should be confirmed through skin scraping [24,25]. This presents resourcing issues, as specialist input can be costly and the diagnostic skills of local health workers can be low. It also presents logistical challenges in the management of pathology testing and patient re-tracing. Both leprosy and crusted scabies are also highly stigmatised, and due to association (both current and historic) with removal from the community setting for treatment, diagnosis can be feared [10,19]. This creates issues with community buy-in, without which screening uptake may be low.

Leprosy is now rare in Australia; it is the only skin NTD to have been endemic in remote Aboriginal communities and eliminated in part, through effective ACD. Historically, ACD in Aboriginal communities was conducted by police, and resulted in forceful removal and isolation of cases from the community [26,27]. During the 1950s-1970s, alongside the availability of effective therapy and a shift to in-community treatment, there was a move towards involving local Aboriginal health workers in ACD to support its cultural appropriateness and community acceptability, as well as to maximise reach in remote geographies [28,29]. Although lessons from historic leprosy control in Australia are informative, this review sought to identify contemporary evidence about effective ACD techniques for leprosy, and to discuss how the findings can be informative for ACD of crusted scabies. In particular, it sought to investigate how ACD campaign design and personnel influence detection rates, and the cost effectiveness of different methods.

## Method

### Literature search

Systematic searches were conducted in MEDLINE, CINAHL, Scopus and the Cochrane Database for Systematic Reviews in October 2019 using a combination of search terms relating to active case detection in concert with the two review diseases. Search results were limited to English language papers from 1999 to 2019. See S1 Appendix for a full list of search terms and results. All search results were exported into EndNote for processing and screening.

### Inclusion and exclusion criteria

To be included in the review studies had to examine an ACD campaign, with one of the two review diseases, leprosy and crusted scabies, as the sole or primary campaign target. Included studies needed to report outcome data on the detection rates of the campaign and a relevant comparison such as local prevalence rate (PR), or the detection rate of a concurrently conducted detection method. These criteria led to the exclusion of papers in which ACD is conducted as part of a control program but the ACD activity is not evaluated against another case detection method and outcome.

### Data extraction, summary and risk of bias assessment

Data were extracted on the ACD campaign setting (community characteristics and country), ACD strategy type, campaign time frame, personnel, method and use of laboratory evidence (typically skin smear) in diagnosis (if not reported recorded as ‘no’). Outcome data extracted were detection and/or prevalence rates (both campaign and comparison). Disease stage at diagnosis was not sought. Due to heterogeneity in campaign type, reporting and setting, a meta-analysis was not performed.

The relevance of the comparative detection or prevalence rate to the study setting impacts the risk of bias in assessment of outcome effect. A significant difference between the campaign and comparator rates will confirm a positive (or negative) effect of ACD in general. However, if the comparator rate has low relevance, the difference may conflate variations in prevalence with campaign effectiveness. For example, comparing the detection rate of a campaign conducted in a high prevalence district to the national prevalence rate in a low prevalence country will inflate the campaign effect size. To assess this risk of bias at the study level, a grading system was developed to rate the relevance of the comparison rate; the grades are identified in the data table (Table 1) and are:

- Low: location and format of low relevance (e.g. national prevalence rate (PR) to campaign new case detection rate (NCDR) in regional sample)
- Moderate: location relevant but comparator rate format and/or timeframe not relevant (e.g. local PR rate to campaign NCDR)
- High: location, timeframe and comparator rate format relevant (e.g. NCDRs of campaigns conducted concurrently and/or in same region).

**Table 1:** Data summary.

First author Country Year	ACD method	Sample	Delivery period	Personnel	Method description	Lab- oratory evidence	Outcomes	Screening accuracy	Comparability to outcome measure
Davoodian [30] Iran 2009	Contact tracing	One large city	Not reported	Screening by leprosy nurses from leprosy clinic Referred for diagnosis at local dermatology centre	Index cases from records one leprosy clinic (1972–2004); skin examination household contacts, education and self-referral neighbours	Yes	NCDR 21.7/10,000 household, 14.3/10,000 neighbour National PR <1/10,000	15% with clinical signs confirmed with laboratory evidence	Low
De Souza Dias [31] Brazil 2007	Community screening, contact tracing	4x100m^2^ zones in one endemic urban municipality	2 weeks per zone	Screening by community and primary healthcare workers Referred for diagnosis in primary healthcare centre under supervision visiting leprologist	Index cases from national registry (1998–2002) geo-referenced for density mapping; door-to-door screening in high density zones	No	Baseline local PR 5.4/10,000; 9.4/10,000 in year of campaign of which 50% identified during campaign	20% suspects confirmed	Moderate
Ezenduka [20] Nigeria 2012	Contact tracing, community screening, traditional healers’ incentive	10 randomly selected communities (5 high prevalence [>1/10,000], 5 low prevalence [<1/10,000]) in two northern states	1 year	Screening by trained health workers and traditional healers Referred for diagnosis is leprosy treatment centre by specialists	Three concurrent programs: 1) Skin examination of household contacts; 2) Rapid village survey consisting mass communication and education campaign and skin examination of self-reporting individuals in public area of village; 3) Skin examination and referral by traditional healers	No	Household contract tracing most cost effective at US$142/case detected, traditional healer incentive US$192/case and rapid survey $313/case; all yielded similar new case numbers	Suspect numbers not reported	High
Fürst [32] Cambodia 2018	Contact tracing	National	4 years	Screening and diagnosis team consisting leprologists from national gov and French non-profit, district and local health workers	Traced and re-screened index cases, household contacts and neighbours to 200m radius; screening, diagnosis and MDT commencement same day by mobile team	No	NCDR higher at household level 25.1/1,000 than neighbour 8.7/1,000 National passive NCDR rate same period 1/10,000	Suspect numbers not reported	Low
Ganapati [33] India 2001	Community screening	Three municipal wards (slums in megacity)	1 month	Youth community volunteers (mixed gender) and supervising paramedicals	Community-wide screening	Yes	Campaign PR 4.2/10,000; state PR 6.6/10,000. 2 cases skin smear positive. US$20/NCD, US$322/skin smear positive	Suspect numbers not reported	High
Gillini [34] Nepal 2018	Community screening	Two high prevalence districts	1 month	Screening by trained local volunteers. Referred for diagnosis at local health centre. Program supervision two Japanese non-profits and WHO	Door-to-door screening	No	Campaign NCDR 5.4/10,000 Local PRs two districts 3.5/10,000 and 2.3/10,000 US$534/additional case compared with PCD.	7% and 10% suspects self-confirmed in two districts. Partial records indicate roughly 50% suspects sought diagnosis	Moderate
Kumar [35] India 2015	Community screening	Scheduled Tribe colonies of one district	2 weeks	Screening by village health nurses and trained volunteers	Door-to-door screening. Suspects brought to health centre for diagnosis by nurse.	No	Campaign community PR 24.6/10,000, pre-campaign community PR 9.8/10,000. District prevalence rate 0.84/10,000. 34% of confirmed cases reported having noticed their skin lesions. 74% treatment completion one-year post campaign.	21% suspects confirmed	Moderate
Mangeard-Lourme [18] India 2017	Contact tracing, community screening	One district	6 months	Leprologist + local health workers; personnel from British non-profit, and trained local health workers.	Index cases identified from leprosy register; contact tracing to household and neighbour levels, community wide screening of Scheduled Castes/Tribes. Suspects escorted for diagnosis at primary healthcare centre by government medical offer and non-profit team.	Yes	PR 37.5/10,000. Local PR 0.73/10,000; ANCDR 13.94/100,000. Community screening of Scheduled Castes/Tribes yielded largest number new cases, and members of these groups consisted 59% all NDCs. 90% of diagnosed new cases commenced treatment at six months post campaign.	100% suspects confirmed	Moderate
Moura [36] Brazil 2013	Contact tracing	Two highest prevalence neighbour-hoods in one endemic municipality of megacity	1 month	4 doctors, 6 med students and 1 nurse	Index cases invited at treatment centres, household and neighbours of accepting index cases invited to participate; Household screening. Suspects referred to healthcare centre for diagnosis.	Yes	Household NCDR 290/10,000, neighbour NCDR 210/10,000 Local PR 3.5/10,000	24% suspects confirmed	Moderate
Pedrosa [19] Brazil 2018	Community screening and contact tracing	277 randomly selected public schools in one city	2.5 years	Trained leprosy technicians	Information and invitation through open seminar, children for whom consent (parents/guardians) obtained received skin examination by trained leprosy technicians at school; suspects and guardians referred to local healthcare centre for diagnosis.	Yes	School screening PR 11.58/10,000 (participants aged <15 years) Contact tracing at household and neighbour level NCDR 357/10,000 Local PR 1.1/10,000 total (0.68/10,000 in children). Grandparents the most common contact (28.6%) identified with current or past leprosy history.	Suspect numbers not reported	Moderate
Rao [37] India 2000	Community screening	Hilly tribal area in one highly endemic state	6 days	Trained (1–3 days) healthcare workers, female community workers and other voluntary workers	Mobile health team met village leaders for cooperation, then conducted door-to-door information/education and screening. Households given visit card which subsequently collected by confirmation team (medical officer and non-profit staff) who performed diagnosis of suspects.	No	NCDR 3.9/10,000 compared with 8.6/10,000 in comparable format campaign with 150 day implementation	4% suspects confirmed leprosy	High
Schreuder [38] Indonesia 2002	Community screening	Two endemic districts on main island	6 months	Mixed gender field workers	Rapid village survey (RVS): school + village information/education and voluntary screening of existing patients, their household contacts, suspects identified by village leaders and any additional self-reporting, suspects subsequently diagnosed by medical officer. Leprosy Elimination Campaign (LEC): information/education and screening of self-reports. Community based diagnosis on clinical symptoms.	No	RVS PR 9.5/10,000, LEC PR 6.4/10,000 Local PR 5/10,000	N/A	High
Shetty [39] India 2009	Community screening	Two areas (one urban, one rural)	5 months + 2 months missed house-holds	Two person health worker teams (local, mixed gender) trained (3 day)	Door-to-door screening. Consent gained from head of household to enter and from individuals before examination. Suspects referred to healthcare centre for diagnosis	Yes	Campaign PR rural 6.72/10,000, urban 2.61/10,000. Local PR rural 1.37/10,000, local PR urban 0.9/10,000	80% rural suspects self-reported, 70% urban suspects. 100% of rural suspects diagnosed, 97% of urban suspects	Moderate
Tiendrebéogo [40] Mali 1999	Community screening	Villages with populations over 1,000 in one health district	2 months	1 doctor, 2 nurses)	Passive and active CD implemented concurrently in randomly selected villages (similar sample size). Passive method: information/education by local nurse, referral of suspects/self-reports to local healthcare centre for examination, then to district healthcare centre for diagnosis by leprosy nurse. Active method: information/education by mobile team (1 doctor, 2 nurses), examination and diagnosis on site.	No	ACD 4.3/10,000, US$72/NCD. PCD (1 year) 1.5/10,000, US$36/NCD. National PR 1.37–2.11/10,000	Not reported	High
Utap [41] Malaysia 2017	Community screening	Three highest prevalence Penan (ethnic minority) settlements	3x1 month	Doctor, medical officers, lab technician with previous health service visits to target communities	Community wide screening. Confirmed cases re-traced by medical officers.	Yes	NCDR 720/10,000 (n = 6/83) Penans PR 5.5/10,000, rest of population PR 0.07/10,000	Not reported	Moderate

It is important to note that assessment against the comparator rate may not be the objective of included studies, but may rather serve as context. There is no evidence that studies examining a single campaign selectively report less relevant comparator rates; when these are used it is assumed to reflect the availability of prevalence data.

## Results

**Fig 1:**
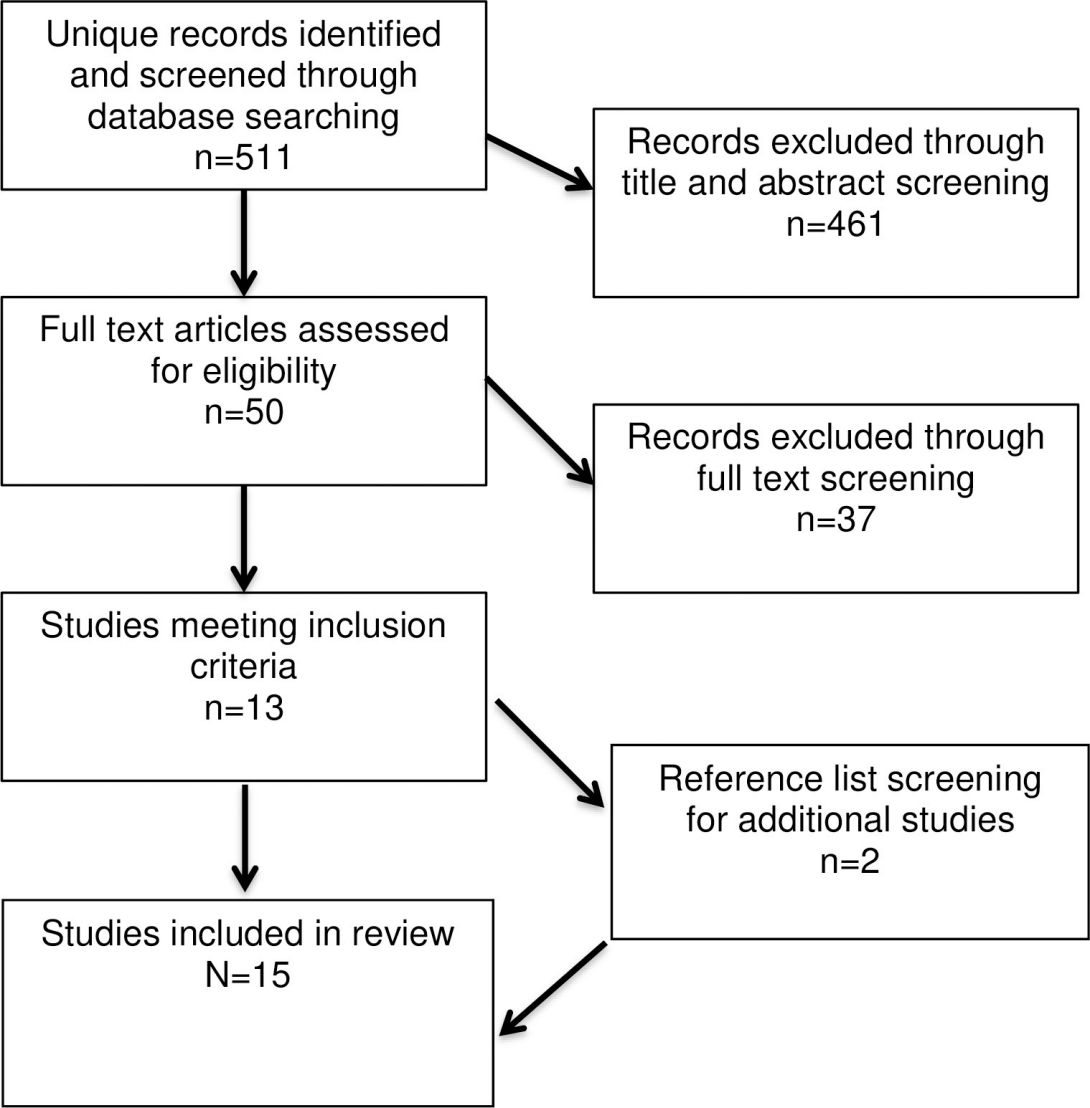
Eligibility flow chart.

The search yielded 511 unique results after removal of duplicates. All papers were screened at the title and/or abstract level and 50 papers were selected for full text review. Of these, 13 met the criteria for inclusion. Reference lists of selected papers were screened for additional resources, which yielded two additional papers, bringing the total included references to 15. This filtering process is presented in Fig 1.

### Study characteristics

All included studies report on ACD campaigns for leprosy. The search yielded only four studies including the term “crusted scabies”, but none met the inclusion criteria. The papers included in the review draw on studies from nine countries, with five from India and three from Brazil (the two countries with largest leprosy numbers globally [17] and the remaining mostly from Asia and Africa). There are no studies from developed countries. Community screening is the most commonly reported detection method, used solely or in concert with contract tracing. Three studies examine contact tracing only.

Not all targeted data extraction points were available in all studies, and extracted data was not uniform. When only detection numbers were reported, the new case detection rate (NCDR) or prevalence rate (PR) (whichever appropriate) was calculated manually. A summary of the included studies is presented in Table 1. Heterogeneity in reporting of both campaign implementation and outcome data inhibit standardization in summation for this review.

### Detection methods

Twelve of the reviewed studies examined community screening methods targeting underserved communities and/or endemic regions. The most commonly reported method of community screening used door-to-door (door-to-door) visits by a small team of community health workers (CHWs) for information and education communication plus screening for clinical signs of leprosy, and referral of positive screened (universally referred to as ‘suspects’) to a local health centre for diagnosis. A number of studies reported alternate settings, including schools and village squares.

Six studies examined contact tracing campaigns, three of which used this method to identify high prevalence areas for targeted community screening. One study used the contact tracing for micro-targeting of geographies for screening, which is achieved by identifying and tracing index patients, then mapping their house location and using case clustering for highly localised community screening [31]. In the included studies, contact tracing was retrospective–identifying index patients through historical national and/or notifiable disease records and seeking out both the index and their contacts to either the household level, and in most cases, the neighbour level [30,31,36].

Less than half of the studies used laboratory testing for diagnosis. The WHO guidance for control leprosy in endemic countries states that diagnosis can be made on the presence of a skin lesion consistent with leprosy with definite sensory loss *or* a positive skin smear [42]. In non-endemic countries, laboratory evidence through skin smear [22] or nucleic acid testing [23] is required. The only laboratory testing reported used in review studies is a slit skin smear sample for microscopy for leprosy bacilli–utilised in half the studies [30,33,39,38].

### Detection personnel

Across studies, screening was typically conducted by a small team (2–4) of community health workers and/or local volunteers/workers. Few studies provide any meaningful detail about the recruitment, training or remuneration (if any) of field or community workers/volunteers, or about roles and responsibilities during the campaign (i.e. information/education, skin examination). When the duration of training is reported, it is typically 1–3 days [e.g.^37^,^39^]. Most studies report whether the gender composition of the team is mixed or single sex. Female community health workers are used in some settings for cultural appropriateness [e.g.^33^,^37^,^39^]. One study reported using local recovered leprosy patients in community screening as a means to enhance community buy-in [33].

Numerous studies reported making contact with community leaders prior to commencing a community based campaign to gain support and raise awareness [18,39,41]. This practice was most common in rural and village based community settings. In a few studies, the relationship (e.g. prior or on-going contact) between mobile health workers and the campaign community is reported [18,41] A minority of studies report whether consent was sought before entering a household or conducting a skin examination. Those that do tend to be more recent, and often involve a developed country implementation partner [e.g.^19^,^39^,^41^].

### Campaign effectiveness

All ACD campaigns in the review studies found new leprosy cases–confirming that routine methods were missing active cases in the sample populations. Heterogeneity in both detection and comparator measures inhibits a meta-analysis of outcomes. Similarly, heterogeneity in the scale, setting and personnel of the reviewed campaigns inhibits a systematic comparative assessment of method design or effectiveness across studies. Data synthesis shows that the highest campaign NCDRs/PRs are in contact tracing campaigns and those conducting community screening of marginalised ethnic groups [18,32,38].

Four studies assessed financial costs. Ezenduka et al [20] compared three ACD methods in Nigeria: household contact tracing, targeted community screening, and a traditional healer incentive to encourage referral to local health centres. The authors found that household contact tracing has the lowest cost per new case detected at US$142/NCD compared to US$192/NCD in the traditional healer incentive and US$313/NCD for community screening. In Tiendrebéogo et al’s [40] study, a community screening ACD campaign (US$72/NCD) cost twice as much per new case detected than PCD (US$36) but yielded a four times higher prevalence rate, and detected cases at an earlier disease stage (the costs/benefits of which are not quantified). Gillini et al [33], reporting on a campaign in Nepal, found a dramatically higher ACD cost than the African studies of US$534/NCD more than the passive method. The baseline cost of the passive method was not reported so total cost is not identifiable. The African studies used local personnel while the Nepalese study involved personnel from WHO and a Japanese non-profit which likely contributed to cost differences. Ganapati et al [33] reported the cost per NCD for a case diagnosed through clinical examination (US$20) was less than 10% of the cost of case diagnosed through skin smear (US$322).

Data synthesis suggest that rapid screening campaigns (<1 month) tend to yield lower rates of suspect confirmation, and find fewer cases than non-rapid methods (typically 6–18 months). When comparing their outcome data with an ACD campaign of similar design and scale but conducted over a much longer time frame (150 days compared with 6 days), Rao et al [37] found that the longer campaign had a NCDR more than twice as high, suggesting rapid survey methods may be less effective. Rapid community screening campaigns were also associated with lower suspect confirmation rates, which is suggestive of high false positive screenings. Seven of the community screening studies, particularly the Indian ones, report both the numbers of positive screened individuals (‘suspects’) and numbers diagnosed. The proportion of suspects confirmed range from 4%-100%, with the average around 10–15%. In the study reporting 4%, a chaser team of medical officers visited the homes of ‘suspects’ identified by community health workers/volunteers meaning 96% of positive screens were false positives. These findings illustrate poor diagnostic capacity amongst community screeners or a low specificity screening test.

In studies relying on ‘suspect’ self-report to local health centres for diagnosis, the proportion of ‘suspects’ who actually attended the health centre is rarely reported. In the few studies that do, attendance rates are roughly 50%. For example, in partial records from the Nepal campaign just under 50% of ‘suspects’ for which this data was captured attended health clinic [33]. In these cases, the difference between suspect numbers and confirmed cases is partly a product of false positives, but also suggestive of barriers to access. The studies with the highest suspect confirmation rates were of longer duration, used more experienced personnel in screening and had campaigns more integrated with local health services [18,39].

Significantly, only two studies reported treatment outcomes. One reported that 74% of cases had completed MDT one-year post campaign [35] and the other reported that 90% had commenced MDT by the end of the six month campaign period [18].

### Barriers and enablers to campaign implementation

Five studies reported barriers to and enablers of ACD campaign implementation. De Souza et al [31] found geographic information systems (GIS) an enabler in a context with few traditional address markers (e.g. street sign/number) in Brazil. Mangeard-Lourme et al [18] found evidence of micro-clustering of leprosy cases in a district in India pointing to the value of geo-mapping for resource allocation/campaign targeting. Numerous studies reported the involvement of community volunteers as an enabler in gaining community support for the campaign [37], including former leprosy patients in one study [33]. However, the use of community workers/volunteers may represent a barrier to campaign effectiveness given low screening accuracy rates.

Lack of transport access, inadequate time for comprehensive screening and long waiting times at the local health centre were reported barriers for both patients and community health workers that led to incomplete coverage of households and attendance of suspects in the Nepalese campaign [33]. One study in India reported that the co-occurrence of numerous chronic skin ailments with leprosy was inhibiting proper diagnosis [39].

Two studies reported findings that evidence how low awareness would inhibit PCD effectiveness; in one Indian study, 45% of newly detected cases had visited a health centre in the past 1 to 2 years, most of which had done so for examinations of lesion(s) specifically [39]. This illustrates poor diagnostic capability in local health services. Another Indian study found that only 34% of newly detected cases reported having noticed their skin lesion(s) prior to diagnosis demonstrating low community awareness which would impede patient self-reporting [35]. Non-availability of MDTs a common barrier to treatment completion [39], and has the capacity to discourage self-reporting if patients do not believe diagnosis will result in effective treatment.

## Discussion

These findings illustrate that ACD campaigns for leprosy yield higher detection rates than routine methods in endemic regions. This higher yield is most significant in contact tracing campaigns, and non-rapid community screening campaigns in highly marginalized, and more geographically remote populations. In two studies in India [18,35] and one in Malaysia [41] reporting ACD campaigns in rural or remote areas dominated by the most marginalised ethnic groups yielded detection rates up to 40–120 times that identified through routine case detection in the same region. This confirms the importance of ACD campaigns in communities facing the highest barriers to healthcare access. These studies also report campaigns using more highly skilled community screeners and due to similarities in context (remote geography and ethnically marginalised population), are the most relevant to crusted scabies detection in the NT of Australia.

Beyond higher detection yields, it is difficult to draw rigorous conclusions about the effectiveness of ACD methods in comparison with PCD methods with limited information about local PCD methods, and few cost effectiveness studies. The lack of cost effectiveness studies is a major limitation on the ability to draw conclusions about ACD campaign effectiveness. It also represents a significant empirical gap given the resource poor contexts in which leprosy occurs. Furthermore, there is inadequate data in this review from which to assess whether ACD campaigns result in better treatment outcomes or overall disease control; an additional significant empirical gap given the resource intensiveness of ACD campaigns.

Heterogeneity in reviewed campaign size and context inhibits systematic comparison of campaign design and effectiveness across studies. Outcome data suggest that contact tracing yields a higher detection rate than community screening. Caution must be used given the small sample size, however, this tentative finding aligns with existing evidence about both contact-based transmission and geographic clustering in leprosy [31,43]—evidence which has been used to mandate contact tracing for notifiable diseases including leprosy and crusted scabies in many countries. In the only ACD comparative cost effectiveness study, contact tracing was also found to be the most cost effective method [20]. All contact tracing ACD campaigns in this review are retrospective, which depends on the existence of a national or notifiable disease register. This is not the case in many countries. In Australia, leprosy has long been a notifiable disease but crusted scabies has only recently become notifiable in a single jurisdiction (NT).

A key weakness of the dominant model of community screening is its reliance on ‘suspect’ self-report to local health centres for formal diagnoses. In relying on ‘suspect’ self-report in ACD, most of the noted barriers to PCD effectiveness will also be similarly prohibitive for ACD. Social stigma and poor healthcare access due to barriers such as limited time, transport and finances will impede self-reporting, and local health services may suffer from poor resource conditions [33]. Additionally, high rates of false positive screening by community health workers may impede suspect self-reporting by creating community skepticism about campaign effectiveness.

Increasing the accuracy of local prevalence rates through ACD can be an end in itself, as this can be used for future resourcing targeting. However, ACD that is not integrated with treatment (both healthcare access and MDT availability) will likely have limited impact on disease control, making resource allocation for this activity questionable. More longitudinal research is needed to assess the impact of ACD campaigns on disease control over time.

### Implications for crusted scabies case detection and review limitations

In summary, the potential impact of ACD on crusted scabies is not quantified from review findings, however, findings show that ACD campaigns tend to find the most missed cases of leprosy in geographically isolated and culturally marginalized communities, which aligns with evidence about ACD more generally [13,14]. This suggests an ACD campaign for crusted scabies in Australia may be worthwhile, given the marginalisation of remote Aboriginal communities and significant barriers to healthcare access amongst this population. The cost effectiveness, particularly for community-wide screening, is not clear given the dearth of cost studies and low applicability of existing ones to the NT. Future cost effectiveness studies in Australia should assess ACD for both simple and crusted scabies in regions where both are endemic. As all studies reported in this review are in developing country contexts and communities with significantly higher population densities than remote northern Australia the transferability of particular models is limited. However, the review illustrates a number of findings that may inform ACD program design in Australia.

As crusted scabies is now a notifiable disease in the NT, contact tracing is a mandated component of disease control making some of the findings from contact tracing campaigns for leprosy relevant. At present, contact tracing is conducted to the household level for new cases of crusted scabies to identify and treat any cases of scabies, both to support scabies control and to ensure the index case does not return to a scabies endemic environment. This review points to the potential value of tracing beyond the household, with cases often found at the neighbour level to index cases in leprosy–a disease less infectious than crusted scabies. This would also align with the high levels of inter-household mobility common in remote Aboriginal communities [44].

The use of historical disease records for identifying index cases may also be of value for ACD in crusted scabies. Similarly to review studies finding historic index cases with active leprosy, crusted scabies cases are often reported as recurrences or on-going due to incomplete treatment [3,10]. Drawing index cases from historical disease records may be a useful starting point for crusted scabies ACD campaign targeting. GIS mapping may also be useful for micro-targeted community screening given the very low population density and the absence of traditional address systems/markers in some remote Aboriginal communities.

A limitation of the literature covered in this review for the Australian context is the reliance on the household as the campaign target. Household centric strategies will miss the most hard-to-reach individuals: the homeless and highly mobile. Recent research found that a high proportion of crusted scabies patients are homeless [10,11]. Any ACD campaign designed for the NT would require consideration of how to capture individuals outside a household system.

There is limited information from which to draw conclusions about the likely acceptability of review ACD programs in Aboriginal communities. Only a few studies discuss the process of consent–an issue that would be highly important in the Australian context. Cultural sensitivity was supported in some studies through the use of community workers/volunteers and making contact with village elders/leaders to gain support prior to community outreach. The involvement of recovered patients is a novel and potentially valuable concept. There is a small but important and highly relevant grey literature about leprosy control in remote Aboriginal communities by the primary clinician operating in the area in the peak endemic period from the 1950s-70s, Dr John Hargrave [27,28,45]. This literature includes a field guide for Aboriginal health workers, whom Hargrave viewed as integral to leprosy control, and discussion of the role of culture in leprosy management, and relationships between communities and healthcare providers.

The review finding of poor screening accuracy by community health workers/volunteers resulting in high numbers of false positives highlights the importance of diagnostic skilling in ACD teams. Lack of healthcare provider awareness and diagnostic skills in low prevalence areas is known to inhibit early and accurate detection of both leprosy and crusted scabies [10,22,46]. There is limited evidence from this review about how diagnostic up-skilling, or laboratory testing and associated patient re-tracing can be managed in leprosy ACDs as only a minority of studies reported it.

The role of new diagnostics needs consideration for both leprosy and scabies ACD. Molecular detection using nucleic acid amplification techniques such as polymerase chain reaction (PCR) to detect M. leprae has been used on skin and nasal swabs [47] and also shows promise for diagnosis of scabies [48]. Point of care diagnostics for scabies are currently under development [49]. As they target detection of scabies mite DNA, these tests are likely to be highly sensitive for crusted scabies and thereby assist health workers in the field with diagnosis of individuals with suspected crusted scabies. Sensitivity is less in ordinary scabies with the much lower mite burden, so a role of POC diagnostic for ACD for scabies in general remains to be determined. Recent innovations in non-invasive diagnostic techniques such as video dermatoscopy [50] may also aide the accuracy of community based screening in the identification of crusted scabies. Increasing use of teledermatology that may also be of benefit to crusted scabies ACD, with even basic photography by relatives being used to support crusted scabies identification in nursing home patients [51] and specialist review of field workers’ photographs of ‘suspect’ crusted scabies cases proving effective in MDA programs [7]. Further research is required to assess the effectiveness of these technological advances in ACD programs.

## Supporting information

S1 PRISMA checklist (DOC)

S1 Appendix Search terms. (DOCX)

S2 Appendix Glossary. (DOCX)
