# Supplementary material for: Active case detection methods for crusted scabies and leprosy: A systematic review
Source: PLoS Negl Trop Dis. 2021 Jul 23;15(7):e0009577. doi: 10.1371/journal.pntd.0009577 (PMC8336788; doi:10.1371/journal.pntd.0009577)
Supplement: S1 Appendix — (DOCX) [file pntd.0009577.s002.docx]

**S1 Appendix. Search terms**

1"crusted scabies" OR "Norwegian scabies"

2 leprosy OR "hansen's disease"

3 “case find*” OR “case detect*” OR “contact trac*” OR “contact screen*” OR “contact investig*” OR “clinical audit” OR “community screen*”

1 AND 3

2 AND 3

1 AND 2 AND 3

|  | 1 AND 3 | 2 AND 3 | 1,2 AND 3 |
| --- | --- | --- | --- |
| CINAHL | 27 | 1 | 28 |
| Scopus | 487 | 3 | 490 |
| MEDLINE | 323 | 3 | 326 |
| Cochrane | 0 | 0 | 0 |
