# Supplementary material for: Active case detection methods for crusted scabies and leprosy: A systematic review
Source: PLoS Negl Trop Dis. 2021 Jul 23;15(7):e0009577. doi: 10.1371/journal.pntd.0009577 (PMC8336788; doi:10.1371/journal.pntd.0009577)
Supplement: S2 Appendix — (DOCX) [file pntd.0009577.s003.docx]

**S2 Appendix. Glossary**

ACD active case detection

CHW community health worker

NCD new case detected

NCDR new case detection rate

MDT multidrug therapy

PCD passive case detection

PR prevalence rate
